# Guillain-Barré Syndrome in Patient With SARS-CoV-2 PCR Positivity Treated Successfully With Therapeutic Exchange Plasma: A First Case Report From Vietnam

**DOI:** 10.3389/fneur.2022.868667

**Published:** 2022-05-25

**Authors:** Sy Duong-Quy, Duc Huynh-Truong-Anh, Thanh Nguyen-Thi-Kim, Tien Nguyen-Quang, Thanh Nguyen-Chi, Quynh Tran-Xuan, Vinh Nguyen-Nhu, Carine Ngo, Timothy Craig

**Affiliations:** ^1^Department of Clinical Research, Biomedical Research Center, Lam Dong Medical College, Dalat, Vietnam; ^2^Department of Intensive Care Unit, Covid-19 Unit of Phu Chanh, Binh Duong General Hospital, Binh Duong, Vietnam; ^3^Division of Pulmonary, Allergy and Critical Care Medicine, Penn State College of Medicine, Hershey, PA, United States; ^4^Department of Expert Consultation, Faculty of Medicine, Pham Ngoc Thach University of Medicine, Ho Chi Minh, Vietnam; ^5^Department of Internal Medicine, Can Tho University of Medicine and Pharmacy, Can Tho, Vietnam; ^6^Department of Family Medicine, Faculty of Medicine, University of Medicine and Pharmacy at Ho Chi Minh City, Ho Chi Minh, Vietnam; ^7^Department of Respiratory Functional Exploration. University Medical Center, Ho Chi Minh, Vietnam; ^8^Department of Pathology, Institute Gustave Roussy, Villejuif, France

**Keywords:** Guillain-Barré syndrome, SARS-CoV-2, COVID-19, MRC scale, therapeutic plasma exchange

## Abstract

Since the first case of Guillain-Barré syndrome (GBS)-associated SARS-CoV-2 (COVID-19) infection reported in 2020, a series of cases have been published in some countries. In this case report, we present a young patient with GBS, whose clinical and laboratory data were appropriate for the diagnosis of GBS due to COVID-19 infection. Neurological examination revealed the muscular weakness of lower limbs with Medical Research Council (MRC) scale of 2/5 associated with diminished reflexes. Laboratory studies showed the positive nasal swab RT-PCR test for COVID-19, leukopenia, increased ferritin and LDH levels, normal electrolyte and liver and kidney function, and normal chest X-ray. The result of cerebrospinal fluid showed the albuminocytologic dissociation. The patient was treated with remdesivir, dexamethasone, anticoagulation, and therapeutic plasma exchange (TPE). Patient's muscle weakness was significantly improved after 1 week of admission. He was discharged at 23rd days of hospitalization and followed-up in the out-patients department.

## Introduction

Since the first COVID-19 patient reported in March 2020, until now, Vietnam has experienced four waves of COVID-19 with more than 2 million confirmed cases and nearly 36 thousand deaths due to COVID-19. During this outbreak of COVID-19 pandemic (September–October 2021), Ho Chi Minh City and Binh Duong Province, located in the South of Vietnam, were two main regions where the number of people who contracted COVID-19 and were hospitalized due to COVID-19 was highest. During this time, the national mortality rate from COVID-19 was 2.4% compared with 1.7% currently.

Although the main symptoms of patients with COVID-19 have been well described previously, COVID-19 patients can present with diverse neurological symptoms such as headache, dizziness, loss of smell and taste, inflammatory polyradiculoneuropathy or Guillain-Barré Syndrome (GBS) (1, 2). Since the first case of GBS-associated SARS-CoV-2 (Covid-19) infection reported by Zhao H. et al. (3), a series of cases have been published (4–9).

Here, we present a patient with GBS, whose clinical and laboratory data were appropriate for the diagnosis of GBS due to COVID-19 infection. The patient was treated successfully by therapeutic plasma exchange (TPE) in combination with standard treatment for Covid-19. This is the first case of GBS associated with the COVID-19 case in Vietnam treated by TPE. It appears that the intervention with TPE made a significant difference compared to most other previously reported patients with Covid-19 induced GBS worldwide during the pandemic outbreak.

## Case Report

An 18-year-old man admitted to an intensive care unit (ICU of COVID-19 Unit of Phu Chanh, Binh Duong General Hospital, Binh Duong Province–Vietnam) during the middle of October 2021 because of fever, cough, sore throat, weakness, and pain of his legs for 4 days. He was otherwise in good health prior to this hospitalization. However, his roommate was diagnosed with COVID-19 and hospitalized two days before his admission. At the time of admission, his temperature was 38·6 °C, pulse was 80/min, respirations were 20/min, blood pressure was 120/80 mm Hg, and SpO_2_ was 95% with room air. He had no symptoms of chest pain or shortness of breath. Neurological examination revealed the muscular weakness of lower limbs with Medical Research Council (MRC) scale of 2/5 (vs 5/5 for upper limbs), diminished reflexes of lower limbs, normal sensation, no abnormal cranial nerve symptoms, and no meningeal signs. The rest of his clinical examination was normal.

Laboratory studies demonstrated a positive nasal swab RT-PCR test for COVID-19, leucopenia, increased ferritin and LDH levels, normal electrolyte and liver and kidney function, and normal chest X-ray. The result of cerebrospinal fluid showed the albuminocytologic dissociation (without an elevation in white blood cells; Table 1). The result of magnetic resonance imaging (MRI) of the spine was normal. The diagnosis of Guillain Barré Syndrome induced by COVID-19 was confirmed in this patient according to the hospital's medical experts. Some other differential diagnosis such as compressive myelopathy, transverse myelitis, and other infectious causes of acute myelitis were also eliminated due to lack of medical history and clinical features.

**Table 1:** Characteristics of laboratory parameters of reported patient.

**Parameters**	**At admission**	**Day 5th–7th**	**After day 7th**	**Institutional normal range**
RT-PCR SARS-CoV-2	Positive	*(non realized)*	Negative	Negative
White blood cell (10^9^/L)	2.66	16.4	10.1	4.0–11.0
*Neutrophil (%)*	37.9	88.07	61.4	45–75
*Lymphocyte (%)*	40·1	6·17	28.1	20–45
Platelet (10^9^/L)	61	325	271	140–500
CRP (mg/dL)	0.26	0.46	0.03	<1·0
Lactate (mmol/L)	2.47	2.13	-	0·5–2·2
Ferritin (ng/mL)	686.3	295.1	256.5	23·9–336·2
Fibrinogen (g/L)	2.67	2.78	0.61	1·5–4·0
TP (%)	99	75	75	>70
APTT (second)	44.5	29.5	15.5	20–40
Arterial Blood Gas				
*pH*	7·41	7.34	7·35	7.35–7.45
*PCO_2_ (mmHg)*	38	35	42	35–45
*${\text{HCO}}_{3}^{-}$ (mmol/L)*	24.1	18.9	23.2	18–23
*Base Excess^−^ (mmol/L)*	−0.4	−6.1	−2.4	−2– +3
*PO_2_ (mmHg)*	93	92	100	80–100
*A-aDO_2_*	9	14	-	5–20
PaO_2_/FiO_2_	442	406	476	
Sodium (mmol/L)	136	135	139	135–145
Potassium (mmol/L)	2.7	3.8	3.8	3.5–5.0
Calcium (mmol/L)	1.11	1.2	1.26	1.1–1.6
Magnesium (mmol/L)	0.83	0.92	0.99	0.73–1.06
Urea (mmol/L)	3.68	5.11	6.15	2.8–7.2
Creatinine (mmol/L)	95.04	70.04	55.69	72–127
eGFR-MDRD (mL/m/m^2^)	95.15	135.32	176.3	≥60
AST (U/L)	46.97	51.47	89.58	0–50
ALT (U/L)	12.81	21.69	77.63	0–50
LDH	351.06	260.53	252.8	<247
Glucose (mmol/L)	4.9	4.5	-	4.1–5.9
Total protein (g/L)	64.4	63.7	-	66–83
Albumin (g/L)	34.7	34.2	-	35–52
Cerebrospinal fluid		*(non realized)*	*(non-realized)*	
Protein (g/L)	0.89	-	-	0.15–0.45
Glucose (mmol/L)	3.65	-	-	NA
Lactate (mmol/L)	1.53	-	-	1.1–2.4
Pandy's test	Positive	-	-	Negative
Cells	0	-	-	0

*CRP, C-reactive protein; LDH, Lactate Dehydrogenase; eGFR, estimated Glomerular Filtration Rate; PT, Prothrombin Time; APTT, Activated Partial Thromboplastin Time; AST, Aspartate aminotransferase; ALT, Alanine aminotransferase*.

**Figure 1:**
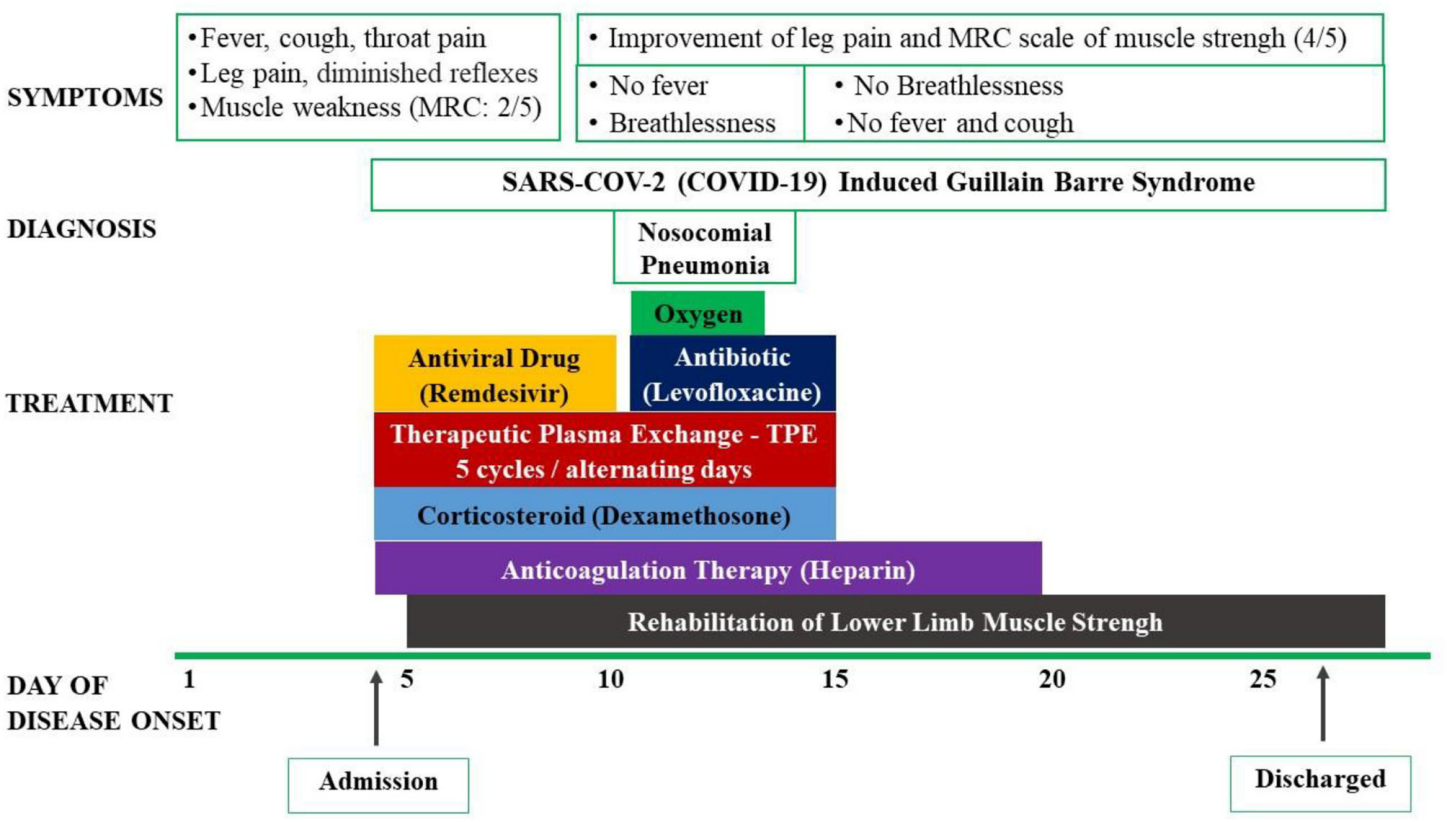
Symptoms, diagnosis, and treatment of reported patient.

**Figure 2:**
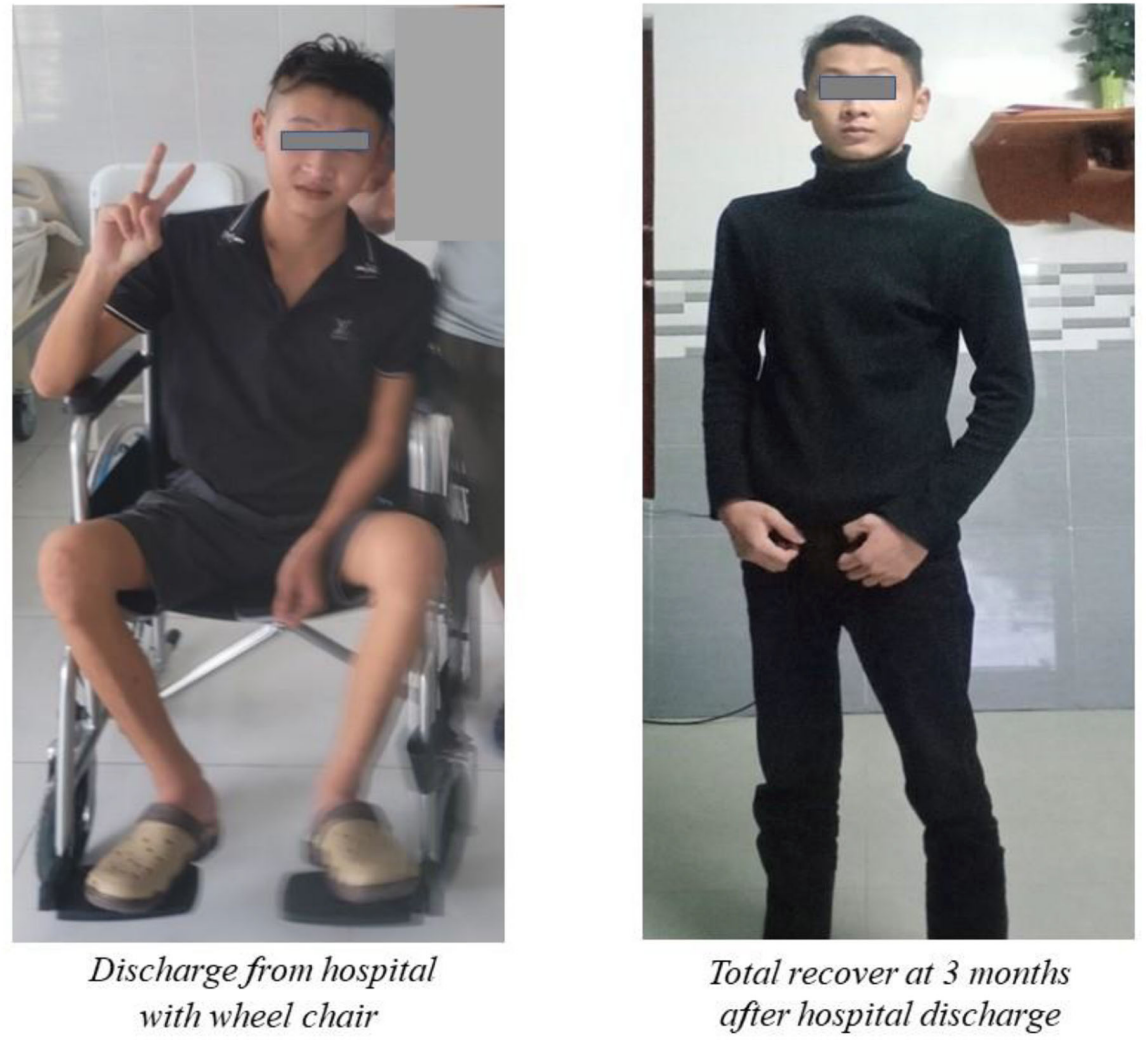
Clinical status of reported patient at post-COVID status.

The patient was then treated with antiviral drug (remdesivir 200 mg loading dose on the first day and 100 mg in the next 4 days), intravenous corticosteroids (dexamethasone 9 mg/day in 3 days and 6 mg/day in the next 7 days), and anticoagulation. He was also treated with the therapeutic plasma exchange (TPE) for 5 alternating day cycles by using 5% human albumin replacement (3.200 ml/cycle). The patient's muscle weakness of the lower limbs improved at day 7 and 14 from 2/5 to 3/5 and then 4/5, respectively, according to the Medical Research Council (MRC) Scale. He also benefited from early rehabilitation to improve muscle strength during his hospitalization in the ICU. At day 7th, he developed a nosocomial lung infection, confirmed by chest X-ray and was treated successfully with short period of antibiotic (intravenous levofloxacin 750 mg/day for 5 days) and conventional oxygen therapy (nasal cannula 3 L/min). His RT-PCR for SARS-COV-2 (COVID-19) were negative at day 14th and 21th. Other laboratory tests were normalized at day 14th. The patient was discharged after 23 days from his hospitalized stay (Figure 1). He has been followed-up regularly at the outpatient Department for Long Covid-19 for his GBS and rehabilitation. At the 3rd month, the patient had a total recover from his GBS with MRC scale of 5/5 and he started to go to work (Figure 2).

## Discussion

Guillain-Barré Syndrome (GBS) is an acute inflammatory polyradiculoneuropathy that results in muscle weakness and diminished reflexes. GBS may occur after immunization or viral infection, including the SARS-COV-2 (COVID-19) (9–14). After one of the first cases of GBS -associated SARS-CoV-2 infection presented by Zhao et *al*. in 2020 (3), other cases have been reported in the world literature during the COVID-19 pandemic (5–8, 10–14).

In 2020, Caress et *al*. analyzed 37 cases of GBS (mean age: 59 years and 65% of male gender), associated with COVID-19 and showed that the mean time from COVID-19 infection to GBS symptoms was 11 days. All 37 patients were free of severe acute respiratory distress syndrome. Albuminocytologic dissociation in cerebrospinal fluid was positive in 76% of cases. Most patients were treated with a single course of intravenous immunoglobulin and improved within 8 weeks. The authors also demonstrated the similar clinical manifestation and severity between non-COVID-19 and COVID-19 induced GBS in the study patients (15).

In 2021, Rumeileh et al. reviewed 73 patients reported in 52 publications, which demonstrated similar results to include the mean age of reported patients being 55 years old and the males accounting for 68.5% of cases (16). In this series of cases, cerebrospinal fluid albuminocytologic dissociation was found in 71 and 100% was negative for SARS-CoV-2. The treatment for GBS in this case series was intravenous immunoglobulin and more than 70% of cases had a good prognosis (16). The first case of GBS with severe acute respiratory distress syndrome induced by coronavirus-2 (SARS-CoV-2) was reported in a 50-year-old male in 2021. In this case report, the patient's cerebrospinal fluid was positive for SARS-CoV-2 (Covid-19), confirmed by RT-PCR (17).

In this case report, we presented a patient with acute progressive and symmetric muscle weakness of lower limbs associated with Covid-19 infection. The results of the clinical examination and laboratory data were appropriate for the diagnosis of Covid-19-induced GBS. It appears to be the first case of GBS associated with COVID-19 reported in Vietnam. Our patient was younger than most other patients in previous reports described above. Besides the treatment with antiviral therapy, anticoagulation and corticosteroids, our patient also received TPE (therapeutic plasma exchange). Although TPE is the treatment of choice for patients with GBS before COVID-19 pandemic, the use of TPE for COVID-19-induced GBS is still rare. The majority of the patients with COVID-19-associated GBS patients noted above were mainly treated with intravenous immunoglobulin. Fortunately, our patient had a total recover from his GBS with MRS scale of 5/5 at 3^rd^ month after hospital discharge.

Both intravenous immunoglobulin and TPE are indicated to treat GBS within 2–4weeks after the onset of neuropathic symptoms to prevent further nerve damage, and both treatments have similar efficacy (16, 18–21). In GBS patients, TPE helps to remove antibodies and other potentially detrimental factors from the blood. TPE might be more effective when starting the treatment within seven days after symptoms onset, but data suggest it is still beneficial in patients treated up to 30 days after disease onset (19).

In addition, TPE is effective in decreasing inflammatory cytokines (including IL-6), acute phase proteins (ferritin, CRP), and improving tissue oxygenation and as such could be considered as an additional therapy to reduce the risk of cytokine storm-induced acute respiratory distress syndrome (ARDS) in COVID-19 patients (22). Moreover, in patients with COVID-19, TPE may help to repair coagulopathy and to restore endothelial membrane by decreasing blood viscosity (20, 23). The association between blood hyperviscosity and the tendency of thrombosis is also considered as a risk of COVID-19 infection.

However, it has been proven in various epidemiological studies, particularly from the United Kingdom, that the incidence of Guillain-Barre syndrome did not increase during the pandemic (24–26). In fact, GBS related COVID-19 is also a rare complication. Definitely, it has been currently more than 10 million people contracted COVID-19 in Vietnam but this case report is the first and only one which has been diagnosed by local experts and declared officially in the country.

One of the limitations of our case report is the lack of electromyography and nerve conduction studies to support the diagnosis of GBS during the acute phase. This was secondary to the lack of access to these tests in our COVID-19 ICU. However, similar to us, we suspect that during the COVID-19 pandemic many health care systems had limited procedures and transportation of patients for testing to decrease health care worker's exposure to COVID-19. In addition, another limitation was also related to the lack of negative results of other infectious test for supporting the diagnosis of GBS during COVID-19 pandemic.

## Conclusion

Guillain-Barre Syndrome (GBS) is an acute inflammatory and demyelinating polyradiculoneuropathy disease. GBS may occur in young patients with COVID-19 as a main symptom of Covid-19 infection. The early management of Covid-19 induced GBS with therapeutic plasma exchange in combination with standard treatment improved the patient's prognosis.

## Data Availability Statement

The original contributions presented in the study are included in the article/supplementary material, further inquiries can be directed to the corresponding author.

## Ethics Statement

Written informed consent was obtained from the individual(s) and/or minor(s)' legal guardian/next of kin for the publication of any potentially identifiable images or data included in this article.

## Author Contributions

The literature search was done by SD-Q, DH-T-A, QT-X, and CN with significant contributions from TN-T-K, VN-N, and TC. Data collection was done by SD-Q, DH-T-A, TN-Q, TN-T-K, and TN-C. All authors contributed equally to analyzing and interpreting the data of case report. SD-Q, QT-X, and DH-T-A drafted the manuscript, with editing by TC and significant contributions by CN, VN-N, TN-C, and CN. All authors contributed to the article and approved the submitted version.

## Conflict of Interest

The authors declare that the research was conducted in the absence of any commercial or financial relationships that could be construed as a potential conflict of interest.

## Publisher's Note

All claims expressed in this article are solely those of the authors and do not necessarily represent those of their affiliated organizations, or those of the publisher, the editors and the reviewers. Any product that may be evaluated in this article, or claim that may be made by its manufacturer, is not guaranteed or endorsed by the publisher.
